# The classification of motor imagery response: an accuracy enhancement through the ensemble of random subspace k-NN

**DOI:** 10.7717/peerj-cs.374

**Published:** 2021-03-02

**Authors:** Mamunur Rashid, Bifta Sama Bari, Md Jahid Hasan, Mohd Azraai Mohd Razman, Rabiu Muazu Musa, Ahmad Fakhri Ab Nasir, Anwar P.P. Abdul Majeed

**Affiliations:** 1 Faculty of Electrical and Electronics Engineering Technology, Universiti Malaysia Pahang, Pekan, Pahang, Malaysia; 2 Innovative Manufacturing, Mechatronics and Sports Laboratory, Faculty of Manufacturing and Mechatronic Engineering Technology, Universiti Malaysia Pahang, Pekan, Pahang, Malaysia; 3 Centre for Fundamental and Continuing Education, Universiti Malaysia Terengganu, Kuala Nerus, Terengganu, Malaysia; 4 Centre for Software Development & Integrated Computing, Universiti Malaysia Pahang, Pekan, Pahang, Malaysia

**Keywords:** Electroencephalography (EEG), Brain-computer interface (BCI), Motor imagery, Random forest, Ensemble learning, Common spatial pattern (CSP)

## Abstract

Brain-computer interface (BCI) is a viable alternative communication strategy for patients of neurological disorders as it facilitates the translation of human intent into device commands. The performance of BCIs primarily depends on the efficacy of the feature extraction and feature selection techniques, as well as the classification algorithms employed. More often than not, high dimensional feature set contains redundant features that may degrade a given classifier’s performance. In the present investigation, an ensemble learning-based classification algorithm, namely random subspace *k*-nearest neighbour (*k*-NN) has been proposed to classify the motor imagery (MI) data. The common spatial pattern (CSP) has been applied to extract the features from the MI response, and the effectiveness of random forest (RF)-based feature selection algorithm has also been investigated. In order to evaluate the efficacy of the proposed method, an experimental study has been implemented using four publicly available MI dataset (BCI Competition III dataset 1 (data-1), dataset IIIA (data-2), dataset IVA (data-3) and BCI Competition IV dataset II (data-4)). It was shown that the ensemble-based random subspace *k*-NN approach achieved the superior classification accuracy (CA) of 99.21%, 93.19%, 93.57% and 90.32% for data-1, data-2, data-3 and data-4, respectively against other models evaluated, namely linear discriminant analysis, support vector machine, random forest, Naïve Bayes and the conventional *k*-NN. In comparison with other classification approaches reported in the recent studies, the proposed method enhanced the accuracy by 2.09% for data-1, 1.29% for data-2, 4.95% for data-3 and 5.71% for data-4, respectively. Moreover, it is worth highlighting that the RF feature selection technique employed in the present study was able to significantly reduce the feature dimension without compromising the overall CA. The outcome from the present study implies that the proposed method may significantly enhance the accuracy of MI data classification.

## Introduction

The process of communication and control in human beings is largely dependent upon the peripheral nerves and muscles. When a healthy individual intends to do something, signals from a specific part of the brain area are sent via the peripheral nerves system to the corresponding muscles, which in turn perform the intended task. Many neurological disorders, which include stroke of the brain, injury to the spinal cord, cerebral palsy, muscle dystrophies, multiple sclerosis and amyotrophic lateral sclerosis amongst others, may impair the regular communication pathways of the signals (Bamdad, Zarshenas & Auais, 2015). If such neural disorders affect individuals considerably, the individuals may partly or generally begin to lose their voluntary motor control. In such scenarios, the individual would not be able to interact by any other means of communication with its surroundings. Researchers are continuously working on a variety of assistive technologies to address these concerns, and it is worth noting that the brain-computer interface (BCI) approach is amongst them. In every BCI system, specific brain signals are converted into control commands for the purpose of handling particular assistive devices (Wolpaw et al., 2002). Amongst the popular BCI applications are mind-controlled wheelchairs, speller, environment control, robotic arm control, biometrics, and emotion recognition (Rashid et al., 2020c). In addition, the BCI technologies are currently being extended from the known traditionally related medical areas to non-medical applications such as virtual reality and games (Rashid et al., 2020c).

Many invasive and non-invasive neuroimaging approaches have been employed to record brain activity. The widely used invasive neuroimaging approaches are intracortical neurone recording and electrocorticography (ECoG). Conversely, the non-invasive approaches are electroencephalography (EEG), single-neuron recordings, magnetoencephalography, functional magnetic resonance imaging, functional near infraRed and positron emission tomography (Rashid et al., 2020d). Based on the recent BCI research activities (Padfield et al., 2019; Guan, Zhao & Yang, 2019), it is evident that EEG and ECoG are the most efficient modalities, so far, for BCI systems. Thus, it is noted that the patterns of mental activity should be decoded in such a way that people can modulate and interpret their thinking in order to deal with a specific BCI technology (Nicolas-Alonso & Gomez-Gil, 2012). In BCI, these signals are considered as control signals, and the broadly utilised neurological control signals are the steady visual evoked potential (SSVEP), the slow cortical potentials (SCP), the potentials evoked by P300, and the signal for motor imagery.

The P300 based BCIs demonstrate comparatively better bit rate without requiring much training process. Nevertheless, the severity of ailment may have a substantial impact on the performance of P300 based BCIs. Although plenty of studies claim that patients with LIS can handle a P300 based BCI for longer periods, the information transfer rate of such patients is still smaller than the healthy users recorded in almost all studies (Rashid et al., 2020d; Lazarou, Nikolopoulos & Petrantonakis, 2018). Besides, some patients are not capable of conducting the experiment due to the higher complexity of stimulating the required response. Furthermore, P300 based BCIs hold a large set of instructions, increasing the trials’ number which decreases the overall performance. The SSVEP based BCI systems also exhibit some drawbacks likewise P300 based BCIs. For example, an extremely weak SSVEP response is generated by a certain group of people, which is very challenging to detect. In spite of lengthy training process, the motor imagery (MI) strategies was able to address the above issues by delivering remarkable outcomes.

Nevertheless, it is worth noting that for the MI-based BCI system, a circumscribed event-related synchronisation/desynchronisation could facilitate the imagination of hand or tongue movement activities (Dai et al., 2019). Hence, it is important to note that the architecture of the BCI system consists of some common steps, namely, data capturing, data preprocessing, feature extraction, classification and device command. Among these steps, feature extraction, selection of the most suitable feature and classification are the most crucial components of any BCI system.

Methodologies for obtaining the best features depend mostly on the form of cognitive impulses used within the BCI and the properties associated with the neural process underlying it. Most of the feature extraction techniques are based on time-domain, frequency-domain, as well as the time-frequency analysis (Padfield et al., 2019). The autoregressive (AR) model and adaptive AR modelling are comparatively effective techniques in the time-domain approach of extracting significant information from the MI responses (Padfield et al., 2019). However, due to the elimination of frequency contents from the signal, the AR model could not extract relevant information always from the MI data. In Batres-Mendoza et al. (2016), the authors proposed a novel function, namely quaternions capable of representing objects in a three-dimensional space in terms of their rotating and directional states. This property may be useful when dealing with multichannel MI EEG data.

In the case of the frequency domain, the widely employed feature for MI classification is the power spectrum (Rodŕiguez-Beŕmudez & Gárcia-Laencina, 2012). It has been reported in the literature that the time-frequency domain-based feature extraction approaches are more superior in comparison to the aforesaid approaches. In such an approach, short-time Fourier transform (Tabar & Halici, 2017), continuous wavelet transforms (CWT) (Ortiz-Echeverri et al., 2019), and discrete wavelet transform (DWT) (Ji et al., 2019) are the most common techniques employed to classify the MI data. Birvinskas et al. (2012) proposed discrete cosine transform (DCT) to extract feature and reduce EEG data size without losing the important information through concentrating the energy of the correlated input data. The preceding investigators (Martisius et al., 2013) introduced wave atom transform (WAT) for EEG feature extraction and dataset reduction without the loss of important signal information. It should be noted that a common spatial pattern (CSP) is a widely recognised feature extraction techniques used for the classification of the MI EEG study (Padfield et al., 2019). There are indeed various types of CSP mechanism, such as common spatio-spectral pattern approach (CSSP) (Kumar, Sharma & Tsunoda, 2019a), common spatio-spectral patterns (CSSSP), sub-band CSP, and regularised CSP (Jin et al., 2019) that are aimed at improving the extraction capabilities of its feature (Padfield et al., 2019).

The importance of feature reduction and extraction cannot be overemphasised as a high-dimensional feature set includes several redundant features or may not be correlated with the BCI-targeted mental states. Hence, feature selection methods should be employed to remove those unnecessary features. Low dimension feature set reduces the possibility of overfitting effects that in turn, yields the enhancement of the classification accuracy. Moreover, a model with lesser features is computationally efficient. Two notable approaches for the selection of features identified are namely filter and evolutionary framework. Filter methods rely on the mutual information between the target variable and each feature (Lotte et al., 2018). Jin et al. (2019) proposed correlation-based channel selection for MI-based BCI. Filter methods have a linear complexity with the number of features. This can, however, lead to a set of redundant characteristics (Lotte et al., 2018).

Conversely, evolutionary algorithms (EA) can provide a potential solution by allowing the selection of features based on optimising the system’s classification accuracy. Different forms of EAs, for instance, particle swarm optimisation (PSO) (Zeugmann et al., 2011), differential evolution (DE) (Qin, Huang & Suganthan, 2009), artificial bee colony (ABC) (Baig et al., 2017), ant colony optimisation (ACO) (Baig et al., 2017) have been applied for MI feature selection. In summary, classification of MI activity for BCI systems is a challenging task due to the low signal-to-noise ratio, non-stationarity nature of EEG, subject dependency and the limited amount of training data (Lotte et al., 2018). Most of the machine learning and deep learning approaches have been employed to classify the MI activity where support vector machine (SVM) and linear discriminant analysis (LDA) were observed to be the widely used (Padfield et al., 2019). The recent studies on MI EEG signal data classification are based on SVM (Jin et al., 2019), dynamic and self-adaptive algorithm (Belwafi et al., 2019), LDA (Suwannarat, Pan-ngum & Israsena, 2018), functional link neural network (Hettiarachchi et al., 2018), Gaussian mixture model (He et al., 2016), sparsity approach (Sreeja & Samanta, 2019), *k*-nearest neighbour (*k*-NN), and Naïve-Bayesian (Bhaduri et al., 2016). Most of these classifiers are primarily constructed for binary classification problems. For classifying multiple MI tasks, these classifiers break into a series of binary classifiers using one-vs-one or one-vs-all strategy. Nevertheless, it is time-consuming to train several binary classifiers when the number of MI tasks is large.

Other than conventional approaches, recently, deep learning (DL) models in which the features and the classifier are learned together directly from data have also been implemented to MI EEG data. A convolutional neural networks (CNN) based multilevel feature fusion model has been proposed in Amin et al. (2019), for MI-based EEG classification. In Lu et al. (2017), an innovative restricted Boltzmann machine (RBM)-based deep learning framework has been introduced for EEG MI classification where the fast Fourier transform (FFT) and wavelet decomposition packages are obtained to train three RBMs. Then, these RBMs are stacked with an extra output layer to construct a frequency deep belief networks (DBN) of four layers. A deep recurrent neural network (RNN) with a sliding window cropping strategy (SWCS) was investigated for classifying the EEG MI signal in Luo, Zhou & Chao (2018). Five class EEG MI data were classified in Nair, Kumar & Mathew (2018), where stack auto encoder (SAE) was used to generate the features, and then softmax layer is utilised for classification purposes. In addition to the aforementioned standalone DL models, researchers attempted to hybridise various DL models in EEG-based BCI studies with promising classification accuracies. Yang, Yao & Wang (2018) put forward a hybrid DL model in which long short-term memory (LSTM) framework was integrated with the CNN for EEG MI classification. Nonetheless, it is worth noting that extensive hyperparameter tunings are often required in DL methods to achieve excellent accuracies. Kumar, Sharma & Tsunoda (2019b) proposed a novel approach known as the OPTICAL predictor that uses a combination of CSP and LSTM network for obtaining improved MI EEG signal classification. Authors in Li & Feng (2019) proposed a new EEG signal classification method to identify the upper limb movements. Initially, the important channels are selected by the random forest algorithm, and then the features are extracted by the wavelet transform. Finally, the features are classified using CNN.

As an alternative to a single classifier, an ensemble of multiple classifiers may be an excellent choice for the purpose of MI classification. Nicolas-Alonso et al. (2014) and Bera et al. (2018) proposed ensemble of LDA and ensemble of extra tree respectively to classify multi-class MI data where the accuracy was not in the satisfactory level. In Chatterjee, Datta & Sanyal (2019), the authors proposed an ensemble approach using a different combination of SVM, *k*-NN and Naive Bayes to classify MI data. Moreover, they employed different architecture of ensemble learning, including bagging, adaboost and loggitboost. Although encouraging accuracies were reported; nevertheless, the models were evaluated with only one dataset.

In order to address the identified gaps, this work sought to provide the following contributions:

- Investigate random forest-based feature selection technique;
- Implement an ensemble of random subspace K-NN algorithm to classify the selected features;
- Conduct a set of experiments validating the effectiveness of the proposed methods.

The rest of the article is organised as follows. “Materials and Methods” explains the proposed methods, description of the datasets and experimental setup. “Results” and “Discussion” presents the results and discussion, respectively. “Conclusions” concludes the article and provides future direction of the research.

## Materials and Methods

**Figure 1:**
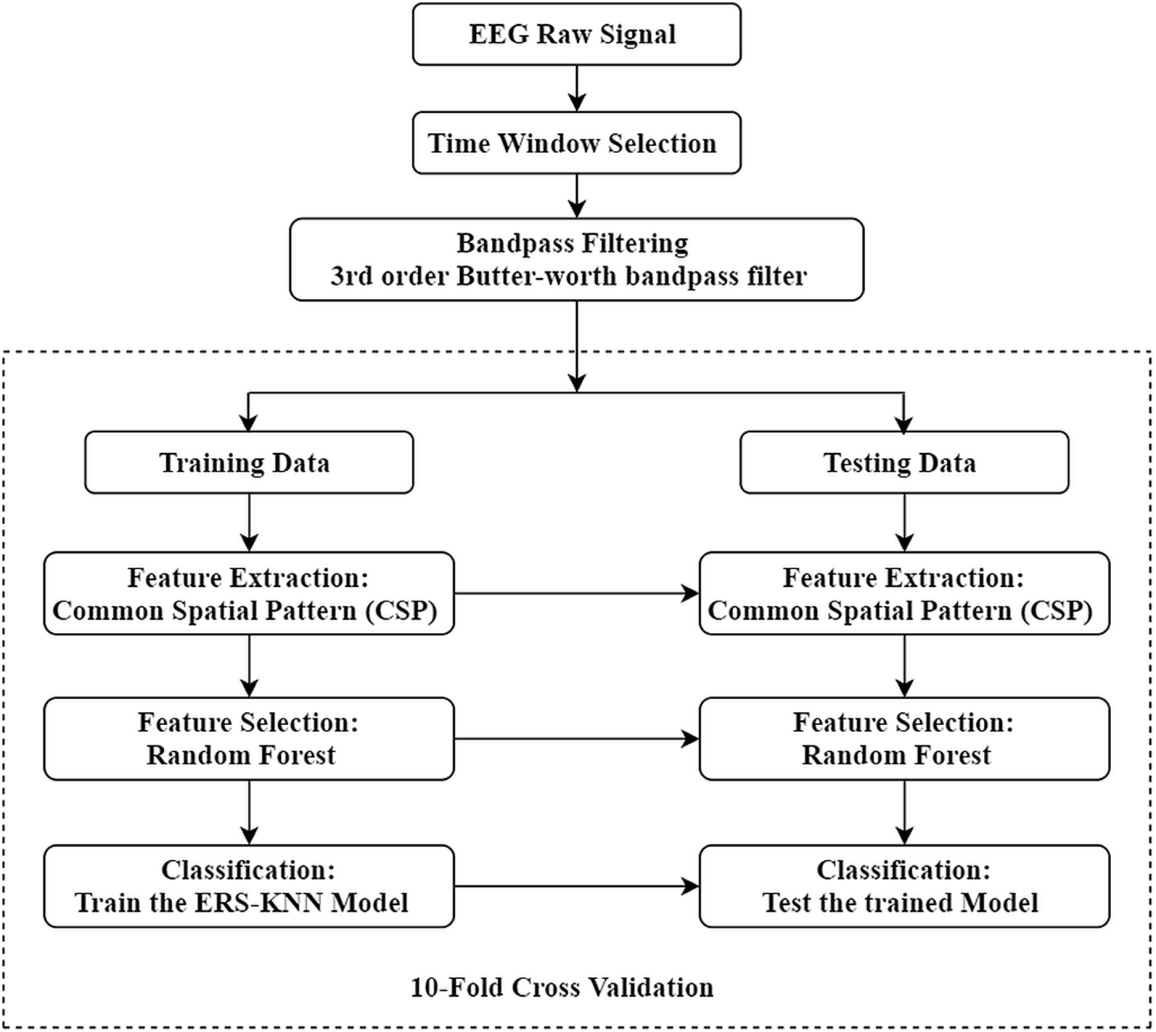
Illustration of the proposed workflow.

The complete workflow of the present study is illustrated in Fig. 1. The aim of this study is to enhance the classification accuracy of binary and multi-class motor imagery data. The experimental EEG and ECoG data have been collected from a publicly available online dataset. For this study, four different datasets from BCI competition have been used to validate the proposed method. Initially, the proper time window of every trial has been selected, and each trial has been filtered between 8 and 30 Hz using a third-order Butterworth filter. The CSP approach has been utilised to extract the feature, and the optimum feature set has been selected through the random forest algorithm. Ten-fold cross-validation has been implemented in this work to avoid overfitting. In other words, the data sets have been divided in such a manner that 10% of the feature vectors are used for testing, and 90% of the feature vectors are used for training in the first iteration. Similarly, in the subsequent iteration, another 90% feature vectors are used for the training set and the rest for the test set. This manner is repeated until all feature vectors have been included in the test set. The extracted feature from training trials has been utilised for training the ensemble of random subspace K-NN. The training model has been tested by the testing trials, and the performance has been evaluated through the different metrics. Finally, the performance of classifier has been assessed under a variety of metrics.

### Details about dataset

Most of the BCI investigations were based on EEG recording using brain wave. There are a few positive features to EEG preference including cost-effective data acquisition device, non-invasive and ease of mobility of data acquisition method. The EEG SNR doesn’t always reach the satisfying point, however. In addition, the EEG analytics algorithm in some cases decreases both the classification accuracy and the rate of data transfer. Electrocorticography (ECoG) is a possible alternative method used in BCIs (Rashid et al., 2020a). While ECoG is an invasive procedure, ECoG’s main advantages are excellent SNR, substantially greater spatial resolution and better classification accuracy (Rashid et al., 2020a). In this study, four motor imagery datasets from ECoG and EEG have been utilised, which are labelled as data-1, data-2, data-3 and data-4, respectively.

**Figure 2:**
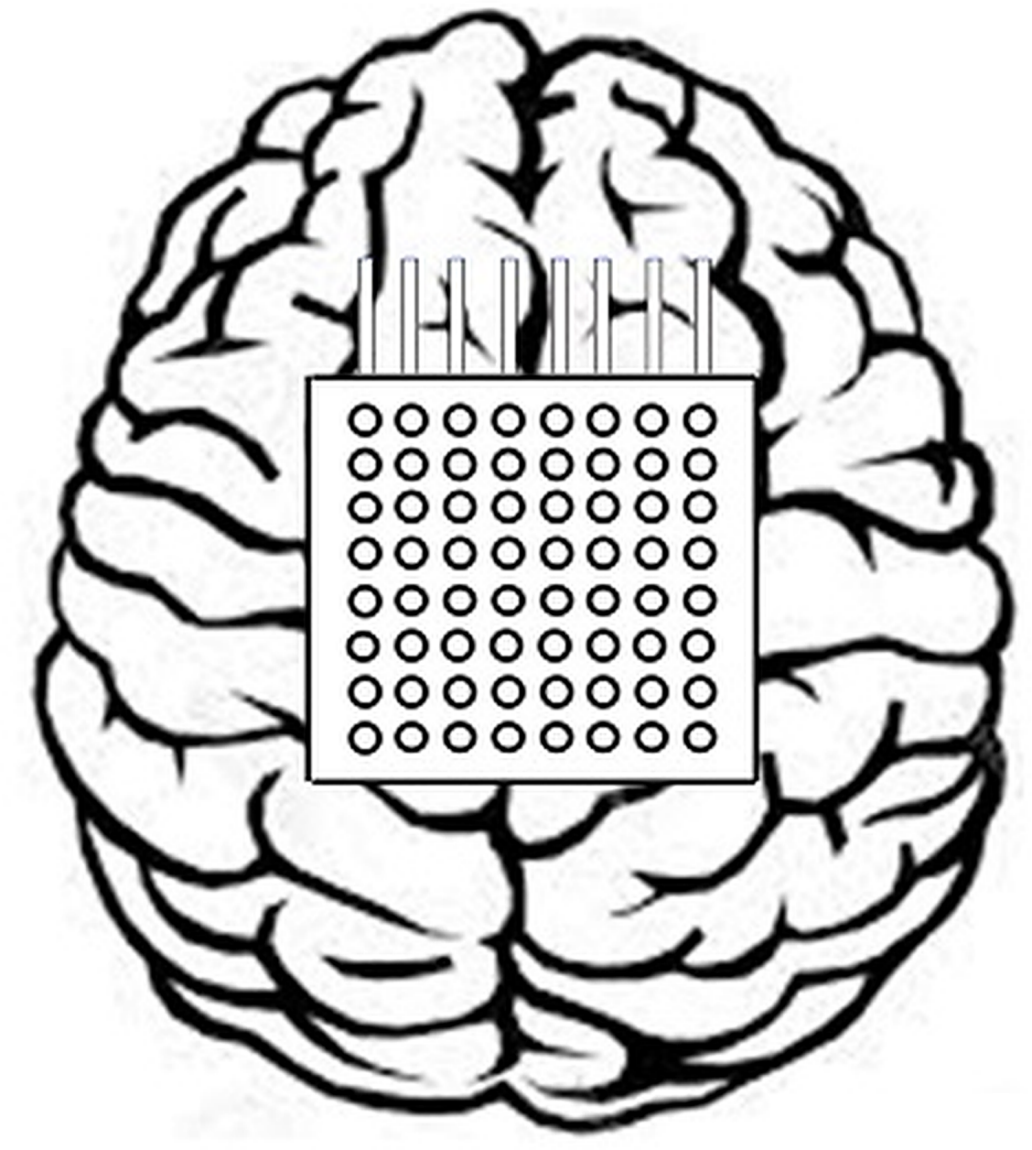
Positioning of the 8 × 8 electrode grid.

The data-1 of this study is the ECoG data which has been picked up from data set I of the 3rd BCI Competition entitled ‘motor imagery in ECoG recordings, session-to-session transfer’ (Lal et al., 2005). This data has been captured from such individual who was affected by epilepsy. The brain activity was recorded when the subject imagined movements of the left small finger or tongue. A total of 64 platinum electrodes are arranged as 8 × 8 electrode grid and placed on the entire right motor cortex area of the brain as shown in Fig. 2.

The ECoG was captured at the sampling rate of 1,000 Hz. After signal amplification, the recorded potentials seemed to be saved as microvolt. This dataset consists of 378 observations (278 training observations, 100 testing observations) where each observation is associated with either of an imagined movement of the finger or of the imagined tongue. The ECoG data were recorded 0.5 s after visual cue ended, and each observation lasted 3 s. This approach was adopted to prevent the influence of unwanted, visually evoked potentials in the ECoG data. The train and test dataset were captured when the subjects experienced different mental states in two distinct periods. The classification of this dataset is comparatively difficult because the data collection was carried out in two distinctive sessions. Thus, the classification algorithm should be capable of classifying certain dataset that has been collected in separate sessions.

**Figure 3:**
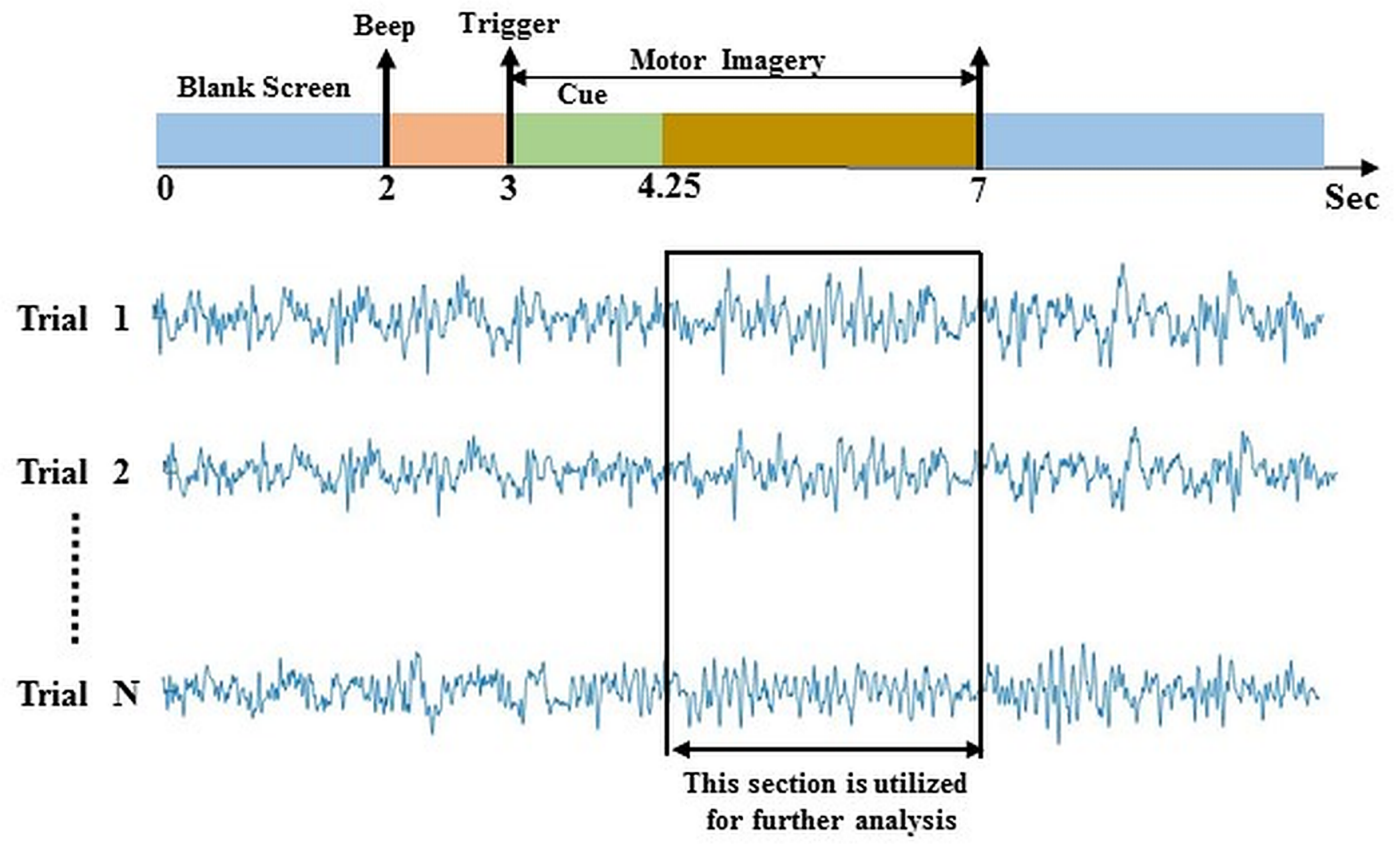
Time window selection of data 2.

Data-2 was taken from the dataset IIIa of BCI competition III (Blankertz et al., 2006). The experimental paradigm is made up of four class EEG motor imagery: right hand, left hand, tongue, and foot. This MI data was captured from three individuals marked as k3b, k6b, and l1b, over 60 channels with a sampling rate of 250 Hz. The data was filtered on Notch filter with the frequency ranges between 1 and 50 Hz. The subject sat in a relaxing chair with armrests, and all the subjects were asked to stimulate the motor imagery activities in accordance with the randomly generated cue. The experiment consists of multiple runs (minimum 6) of 40 trials each; after the beginning of the trial, the first 2 s were quite, at *t* = 2 s an auditory stimulus symbolised the beginning of the trial; then from *t* = 3 s, a left, right, up or down arrow was demonstrated for 1 s; at the same time, the participant was instructed to generate left hand, right hand, tongue or foot movement imagination respectively until *t* = 7 s illustrated in Fig. 3. In this study, data from 4.25 s to 7 s of each trial has been used for further analysis. In a randomised order, each of the four cues was illustrated ten (10) times within each run.

The data-3 used in this experiment is from BCI competition III, dataset IVa provided by the Fraunhofer FIRST, Intelligent Data Analysis Group (Dornhege et al., 2004). The EEG signal was recorded from five healthy subjects denoted by ‘aa’, ‘al’, ‘av’, ‘aw’, and ‘ay’. The EEG data consists of two classes (right hand and right foot) are recorded using 118 channels. A total of 280 trials for each subject was recorded. The sampling rate of the data is 1,000 Hz, which was down-sampled to 100 Hz for analysis purposes.

In data-4, we have made use of publicly available BCI competition IV dataset 2A (Brunner et al., 2007). It contains EEG signals from 22 EEG channels and 3 EOG channels with left mastoid as a reference, while performing multiple MI tasks: right hand, left hand, foot, and tongue movements. The dataset contains nine healthy subjects, and each subject has two sessions, one training session and one test session. Each session has 288 trials of MI data with 72 trials for each MI task. The EEG signals were sampled at the sampling rate of 250 Hz.

### Common spatial pattern

The most popular and well-known feature extraction technique in MI-based BCI studies is CSP which has been employed in this study. The CSP approach provides spatial filters that are capable of maximising the variance of one class while simultaneously reducing the variance of the other class (Ramoser, Müller-Gerking & Pfurtscheller, 2000; Rashid et al., 2020b). The standard CSP is only powerful in differentiating the two classes. In Dornhege et al. (2006), a multi-class paradigm using CSP was proposed, which was the enhancement of standard CSP. Here, the concept is to split the k-class problem into a set of k binary classes and to distinguish each class against the rest of the classes, which is known as the one vs rest approaches. The normalised spatial covariance matrix of an EEG trial can be computed as follows:

(1) $${C_{class}} = \displaystyle{{A{A^T}} \over {trace\left( {A{A^T}} \right)}}$$

where A refers a trial that is [Ch × S] matrix (Ch is the total channel number and S is the sample number) and ${\rm \; }class$ is the types of MI activity in the dataset. The trace $trace\left( {A{A^T}} \right){\rm \; }$ is the summation of the diagonal elements of matrix $A{A^T}$. The mean normalised covariance of each group can be computed by doing average over all trials of each group. The composite spatial covariance can be expressed by

(2) $${C_r} = \mathop \sum \limits_{class = 1}^m {\bar C_{class}}$$

where, *m* denotes the group number in the MI data. The decomposition of a trial S was computed using the projection matrix as follows:

(3) $$X = SL$$

As the total corresponding eigenvalues seems to be one, the variances of the first and last rows of X are perfect features for classification. We used the variances of both the first and last rows as features in this study. The variance was determined according to:

(4) $${V_r} = \displaystyle{{\sum {{\left( {{X_R} - \overline {{X_R}} } \right)}^2}} \over {Z - 1}}$$

where, ${X_R}$ is a row of X and Z is the length of this row.

### Decision tree and random forest

Decision tree (DT) is a supervised machine learning model that can be used in both classification and regression problem. The most successful methods of DT induction called classification and regression trees (CART) proposed by Breiman et al. (1984). The algorithm is supposed to non-parametric and generates binary trees from such data which can be explained by the discrete and continuous features (Grabczewski, 2014). In CART, information gain, Gini diversity index (GDI) and gain ratio are used to split the attributes. In information theory, the predicted information gain value of one random variable in a pair (X, Y) is acquired through observation of another variable. Calculating information gain is based on the theoretical concept of entropy, which is defined by the Eq. (5) for a given variable X as (Haq et al., 2019);

(5) $${\rm H}\left( {\rm X} \right) = - \mathop \sum \limits_{{\rm i} = 1}^{\rm n} {\rm P}\left( {{{\rm x}_{\rm i}}} \right){\log _2}\left( {{\rm P}\left( {{{\rm x}_{\rm i}}} \right)} \right)$$

The GDI is utilised to calculate the purity of potential child nodes in order to optimise the average purity of two child nodes during separation. The GDI of a node is represented by Eq. (6)

(6) $${\rm GDI} = 1 - \mathop \sum \limits_{ i} {{\rm P}^2}\left( { i} \right)$$

where, the sum is over the classes i at the node, and *p*(*i*) is the observed fraction of classes with class *i* that reach the node.

Random forest (RF) is one of the renowned ensemble learning-based machine learning strategy, where many decision trees (DT) are aggregated as the single DT being prone to overfit. Even though RFs are recognised computationally intensive, due to its non-parametric nature and the ability to manage high-dimensional data, it has attracted considerable attention (Haq et al., 2019). Due to their predictive ability, RFs often provide useful internal estimates of error, correlation, variable importance, and strength. Gini and permutation value are the two widely used predictor importance measures. Permutation importance is deemed to be more accurate and is determined by adjusting the prediction error when any correlation between the target variable and the predictor concerned is reduced by permuting the predictor values. Less relevant variables will result in little to no change in the error of prediction, while higher important variables will result in a more significant change. As mentioned in Janitza, Celik & Boulesteix (2018), variable importance is determined by comparing the error of prediction before and after a variable’s values have been permutated.

(7) $${\rm V}{{\rm I}_{\rm i}} = \displaystyle{1 \over {{\rm ntree}}}\mathop \sum \limits_{{\rm t} = 1}^{{\rm ntree}} \displaystyle{1 \over {\left| {{\rm OB}{{\rm B}_{\rm t}}} \right|}}\mathop \sum \limits_{{\rm i} \in {\rm OO}{{\rm B}_{\rm t}}} \left\{ {{\rm E}\left( {{{\rm y}_{\rm i}} \ne \hat {\rm y}_{{\rm it}}^{\rm *}} \right) - {\rm E}\left( {{{\rm y}_{\rm i}} \ne {\hat {{\rm y}}_{{\rm it}}}} \right)} \right\}$$

where E(.) denotes the error estimation function, OOBt denotes the set of indices of observations which are out-of-bag for tree ${\rm t} \in \left\{ {1, \ldots \ldots ,{\rm ntree}} \right\}$ and ${\hat {\rm y}_{{\rm it}}}$ and $\hat {\rm y}_{{\rm it}}^{\rm *}$ denote the predictions by the *t*-th tree before and after permuting the values of variable Xj, respectively.

The procedure for the estimation of OOB predictor importance values by permutation is shown in Table 1. Suppose that R is a random forest of T learners and p is the number of predictors in the training data.

**Table 1:** Algorithm for OBB predictor importance by permutation.

Algorithm: OBB Predictor Importance by Permutation
For tree t, t = 1……T Identify the OBB observations Identify The indices of the splitting predictor variables, ${s_t} \subseteq \left\{ {1 \ldots p} \right\}.$ Calculate the OBB error ${\varepsilon _t}$. For each predictor variable ${x_j},{\rm \; }j \in {s_t}$ Randomly permute the observations of ${x_j}$. Estimate the model error, ${\varepsilon _{tj}}$ using the OBB observations. Estimate the error difference ${d_{tj}} = {\varepsilon _{tj}} - {\varepsilon _t}$ At ${d_{tj}} = 0,$ the predictor variables are not split. For every predictor, calculate the mean, $- {d_j}$ and standard deviation, ${\sigma _j}$ of the error differences over the learners.The OBB predictor importance is given by $-d_j/{{\rm \sigma}_j}$ for ${x_j}$.

### The ensemble of random subspace *k*-NN

The employment of ensemble-based learners has received due attention in recent times as such a technique enhances the basic learner’s performance and capability. Ensemble learning is the process of combining different classification techniques to build a powerful composite model from the data. The purpose of this approach is to obtain a greater prediction rate from several models than any model can on its own. The most generally utilised ensemble strategies are bagging, adaboost, and random subspace. An ensemble inducer can be of any sort of base classifiers such as *k*-nearest neighbour (*k*-NN), decision tree, and other types of base learner algorithm. In this research work, the *k*-NN and random subspace have been used as the base learner and ensemble strategy, respectively.

**Figure 4:**
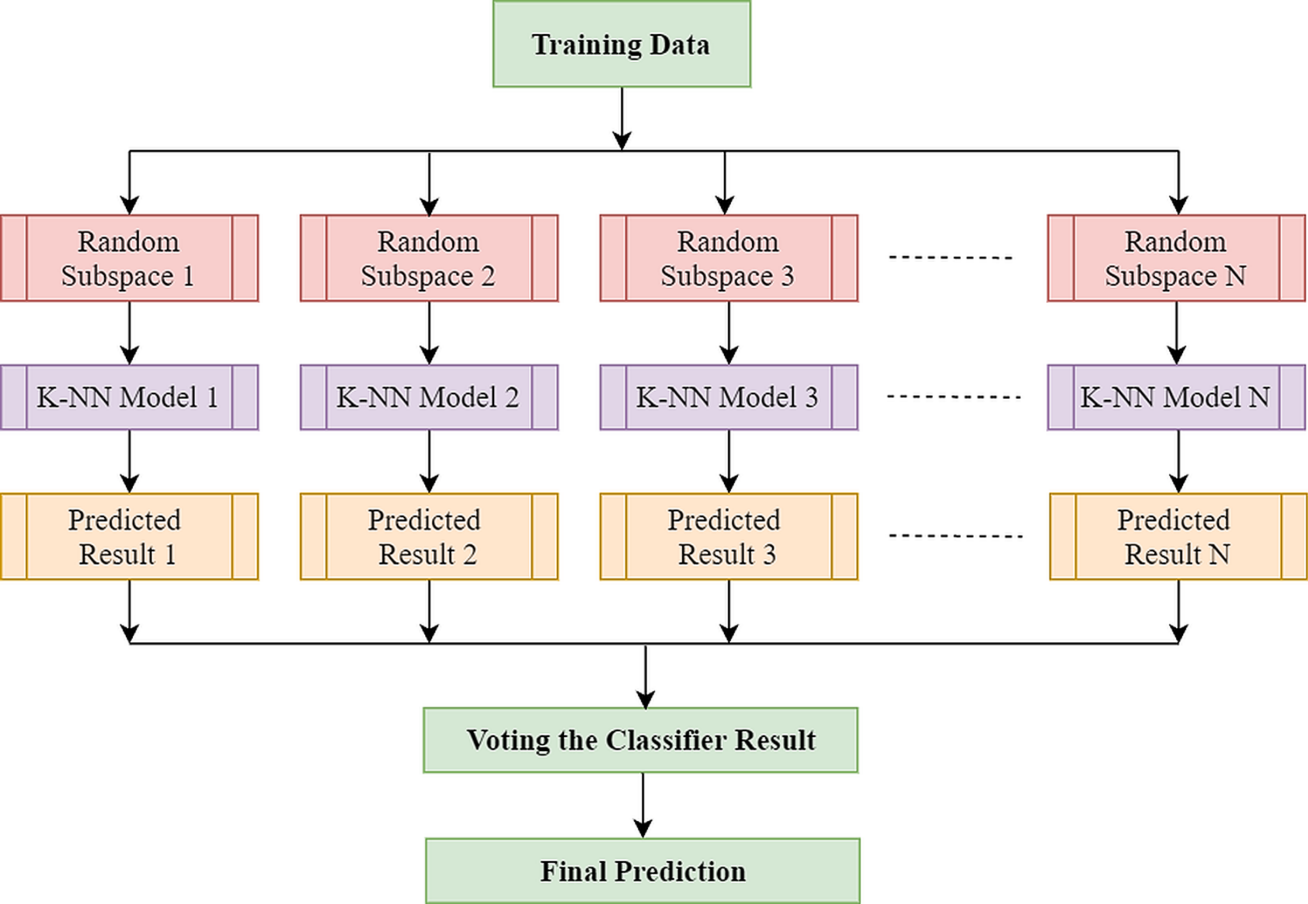
Architecture of random subspace-based ensemble *k*-NN.

The *k*-NN algorithm is a basic technique of machine learning in which the features corresponding to the various classes will form individual clusters in the feature space. To classify a test feature vector, this classifier takes into account the *k* distance metric between the test sample features and those of the nearest classes. The number of neighbours and the types of distance metrics are the main factors in *k*-NN architecture. The *k*-NN is widely used in pattern recognition because of its strong generalisation and simple implementation (Sharon et al., 2019). However, EEG’s high dimensionality usually hampers *k*-NN efficiency. The complexity of these characteristic spaces increases exponentially with the number of features (Sharon et al., 2019). In such a case, an approach that may leverage the advantages of *k*-NN classifier without being adversely affected by the sparsity of high-dimensional data would be highly favoured, and the well-known ensemble learning technique effectively takes advantage of high-dimensionality (Ho, 1998a). The ensemble classifier builds a robust classifier by combining the outcome of some weak or base classifiers to improve the overall classification efficiency. *k*-NN is stable to adjust the training datasets while being prone to feature sets variation (Chaudhary et al., 2020). As *k*-NN is perceptive to input choices, random subspace-based ensemble systems are competent to enhance the efficiency of single *k*-NN classifiers (Ho, 1998b). Random subspace is one of the most widely employed ensemble techniques which creates individual classifiers using subspaces of features picked randomly. Furthermore, eventually, the results of each independent classifier are combined using a standard majority vote to yield the final result. The complete process is illustrated in Fig. 4. In the case of *k*-NN classifier, only the selected features are inputted to the distance when a test sample is selected as a prototype. Yet in subspace *k*-NN, it is the projection of all points to the chosen subspace, and it calculates the *k* neighbouring neighbours using the distances. Once a random subspace is selected, a new set of *k*-nearest neighbours is determined. The majority voting on the test sample’s class membership is achieved by combining k adjacent neighbours in each subspace chosen. In this ensemble, the same training sample that reoccurs if it is found to be in multiple chosen subspace in the centre of adjacent *k* neighbours.

### Performance evaluation

The performance of the intended approach has been assessed by confusion matrix, classification accuracy, precision, sensitivity, specificity, recall and F1-score. The classification accuracy (CA) is computed by Eq. (8)

(8) $$\rm CA = \displaystyle{{TP + TN} \over {TP + FN + TN + FP}} \times 100\%$$

where TP = true positive, FN = false negative, TN = true negative and FP = false positive. Moreover, the formulas for precision, sensitivity, specificity, recall and F1-score are shown in Eqs. (9)–(13) respectively.

(9) $$\rm Precision = \; \displaystyle{{TP} \over {TP + FP}} \times \; 100\%$$

(10) $$\rm Sensitivity = \; \displaystyle{{TP} \over {TP + FN}} \times \; 100\%$$

(11) $$\rm Specificity = \; \displaystyle{{TN} \over {TN + FP}} \times \; 100\%$$

(12) $$\rm Recall = \; \displaystyle{{TP} \over {TP + FN}}$$

(13) $$F1 = \; \displaystyle{\rm {2*Precision*Recall} \over {\rm Precision + Recall}}$$

## Results

In this study, the efficacy of a variety of machine learning-based classification algorithms including LDA, Naïve Bayes, SVM, *k*-NN, RF and random subspace *k*-NN (ERS-*k*-NN) were investigated. As for the Naive Bayes and SVM, the Gaussian kernel is used. Conversely, for the *k*-NN, the number of neighbours, *k* selected is ten (10) whilst the Euclidean distance metric is used. For the RF classifier, the number of learners and the maximum number of splits employed are 30 and 359, respectively. Table 2 shows the classification accuracy of these algorithms with respect to the data-1, data-2, data-3 and data-4. All the classifiers have achieved comparatively better accuracy with data-1. In data-1, the lowest accuracy, which is 95.8% has been observed by the LDA, whereas the highest accuracy, 99.21% has been recorded by the ERS- *k*-NN. In data-2, it is evident that the accuracy gaps between the evaluated classifiers are rather wide. The lowest and highest average accuracy are found to be 61.93% and 93.19%, which have been achieved by the LDA and ERS- *k*-NN, respectively. A similar observation can be seen for data-3, in which the LDA and ERS- *k*-NN also have been contributed to the lowest (68.26%) and highest classification accuracy (93.57%), respectively. Nonetheless, in the case of data-4, the worst accuracy (75.78%) is observed when Naïve Bayes was employed. However, it is evident that the ERS- k-NN classifier has obtained the best accuracy (90.32%).

**Table 2:** The classification accuracy of the implemented machine learning-based classification algorithms.

Metrics	Data-1	Data-2				Data-3						Data-4									
		k3b	k6b	l1b	Mean ± SD	aa	al	av	aw	ay	Mean ± SD	A01	A02	A03	A04	A05	A06	A07	A08	A09	Mean ± SD
LDA	95.8	82.8	36.3	66.7	61.93 ± 23.61	67.9	68.2	70.6	69.4	65.2	68.26 ± 2.01	93.6	87.1	90.8	81.4	61.4	64.1	56.3	86.2	82.2	78.12 ± 13.81
Naïve Bayes	97.1	91.1	73.3	94.2	86.20 ± 11.27	89.3	73.9	82.9	78.2	76.6	80.18 ± 6.05	90.2	85.6	89.1	83.2	58.4	60.2	47.4	88.4	79.6	75.78 ± 16.05
SVM	98.1	91.4	57.5	82.1	77 ± 17.51	93.6	90.4	88.4	89.4	86.4	89.64 ± 2.66	96.8	91.2	93.4	88.4	64.6	78.6	62.4	90.6	84.2	83.35 ± 12.42
*k*-NN	97.5	88.1	57.9	86.3	77.43 ± 16.94	89.6	88.6	86.8	90.2	87.6	88.56 ± 1.39	92.6	88.4	90.1	81.4	69.4	70.1	59.1	79.8	77.1	78.66 ± 11.02
RF	96.4	86.4	76.3	83.8	82.16 ± 5.24	91.8	92.2	89.4	88.6	86.2	89.64 ± 2.45	89.2	90.2	92.6	88.1	60.8	59.6	59.8	88.2	79.6	78.67 ± 14.39
ERS- *k*-NN	99.21	98.33	83.33	97.92	93.19 ± 8.54	94.64	94.64	95.00	93.21	90.36	93.57 ± 1.9	98.96	97.57	97.57	93.75	80.21	88.54	71.18	94.79	90.28	90.32 ± 9.24

**Figure 5:**
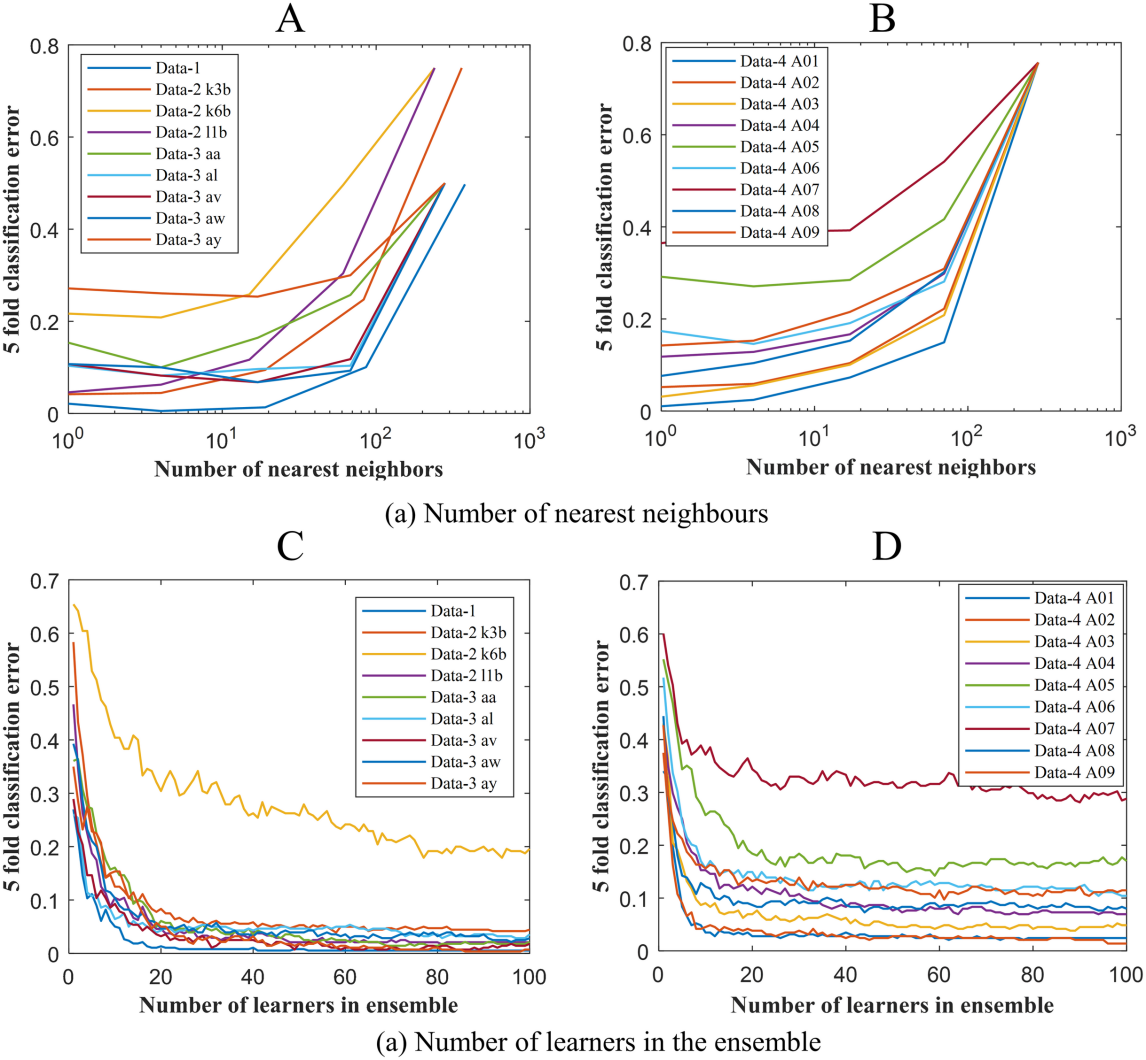
Classification error vs (A) the number of nearest neighbours for Data-1, Data-2 and Data-3 (B) the number of nearest neighbours for Data-4 (C) the number of learners in the ensemble Data-1, Data-2 and Data-3 and (D) the number of learners in the ensemble Data-4.

**Figure 6:**
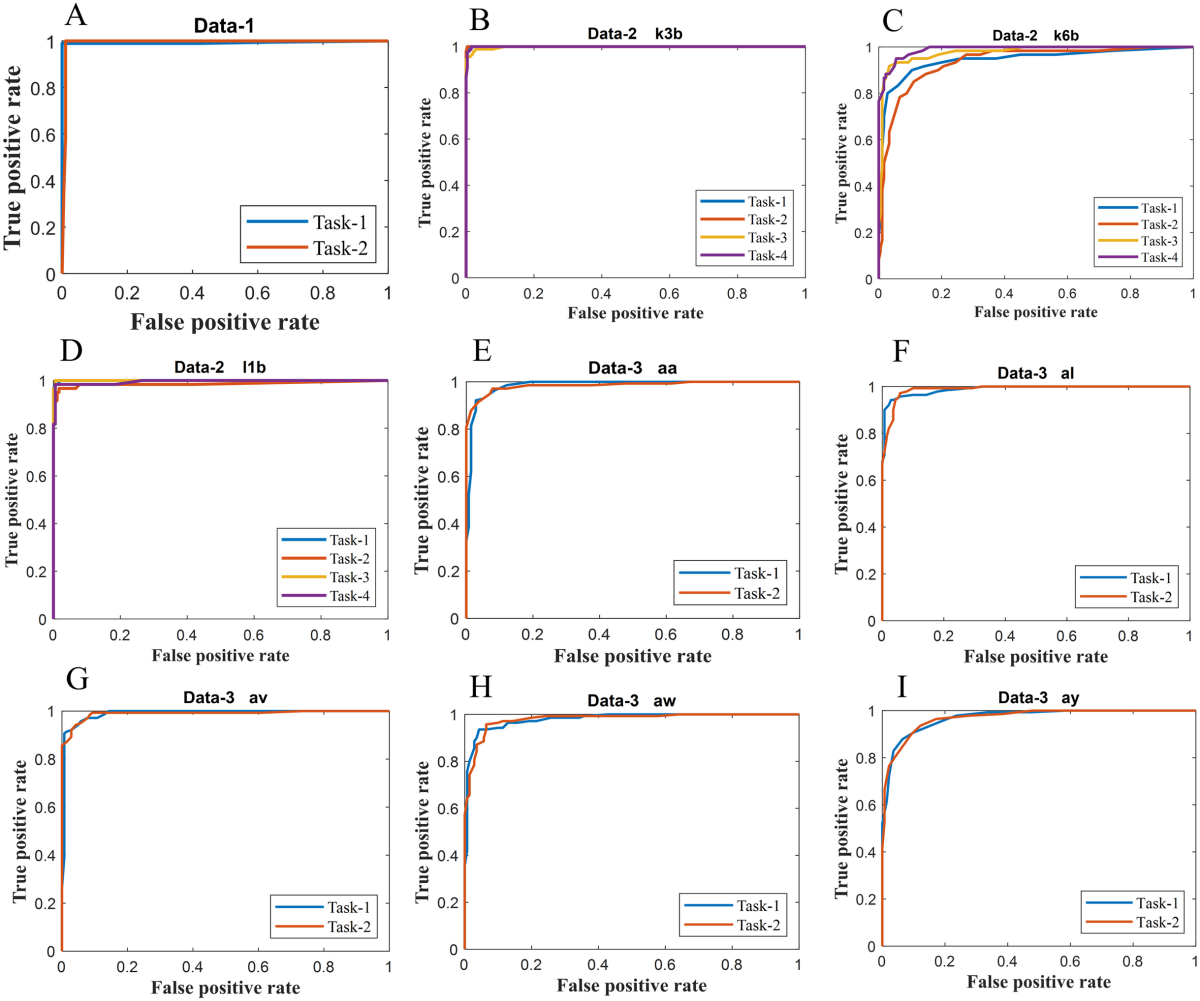
ROC of ERS-KNN classifier for (A) Data-1 (B) Data-2 k3b (C) Data-2 k6b (D) Data-2 l1b (E) Data-3 aa (F) Data-3 al (G) Data-3 av (H) Data-3 aw and (I) Data-3 ay.

To get the optimum performance from ERS- *k*-NN, a good choice for *k*, that is the number of nearest neighbours must be selected. In this study, the value of *k* has been chosen via the cross-validation technique on an evenly spaced logarithmic scale. Figure 5A shows the variation of classification error with *k*. The selection of leaners set in the ensemble is another crucial parameter of any ensemble-based classification algorithm. It is highly desirable to utilise the smallest number of learners in the ensemble that still give good classification. There seems to be no advantage in an ensemble with more than 50 learners. In this study, the ensemble has been constructed with 50 *k*-NN learners. Figure 6B shows the variation of classification error with the number of learners in the ensemble.

We have further assessed the trained ERS- *k*-NN, model through a wide range of performance evaluation metrics including sensitivity, specificity, false-positive rate (FPR), error rate, kappa, precision, F1-score, MCC, AUC and execution time. Table 3 shows the performance of ERS- *k*-NN classifier in terms of different performance evaluation metrics. From Table 3, it is noticeable that the performance of ERS-*k*-NN, when employed in data-1, is comparatively much higher than the data-2, data-3 and data-4. For example the sensitivity and specificity for data-1 are 99.47% and 98.94% respectively, whereas these percentages are 93.19% and 97.73% respectively for data-2. Similarly, the precision and F1-Score for data-1 are 0.98 and 0.99, respectively, whereas, for data-2, these values are 0.93 and 0.926, respectively. The performance of data-2 is lower due to the poor performance of subject k6b. The accuracy of k6b using ERS- *k*-NN is only 83.33%, and this accuracy contributes to drop the overall accuracy. In the case of data-3, the first three subjects (aa, al and av) have achieved excellent accuracy, but the accuracy of subject ay is dropped, which contributed to the reduction of the overall accuracy for data-3. A similar observation is also noticeable for data-4, the subjects (A01, A02, A03, A04 and A08) presented very encouraging accuracy, but the accuracy from the remaining subjects (A05, A06, A07 and A09) was desirable enough, which in turn, resulting in the reduced overall accuracy. The overall classification accuracy for data-3 and data-4 are 93.57% and 90.32%, respectively.

**Table 3:** Performance of ERS- *k*-NN based classification algorithms.

Metrics	Data-1	Data-2				Data-3						Data-4									
		k3b	k6b	l1b	Mean ± SD	aa	al	av	aw	ay	Mean ± SD	A01	A02	A03	A04	A05	A06	A07	A08	A09	Mean ± SD
Accuracy (%)	99.21	98.33	83.33	97.92	93.19 ± 8.54	94.64	94.64	95.00	93.21	90.36	93.57 ± 1.9	98.96	97.57	97.57	93.75	80.21	88.54	71.18	94.79	90.28	90.32 ± 9.24
Sensitivity (%)	99.47	98.33	83.33	97.92	93.19 ± 8.54	92.14	90.00	95.71	90.00	90.71	91.71 ± 2.39	98.96	97.57	97.57	93.75	80.21	88.54	71.18	94.79	90.28	90.32 ± 9.24
Specificity (%)	98.94	99.44	94.44	99.31	97.73 ± 2.85	97.14	99.29	94.29	96.43	90.00	95.43 ± 3.52	99.65	99.19	99.19	97.92	93.40	96.18	90.39	98.26	96.76	96.77 ± 3.08
FPR (%)	1.06	0.56	5.56	0.69	2.27 ± 2.85	2.86	0.71	5.71	3.57	0.10	2.59 ± 2.26	0.35	0.81	0.81	2.08	6.60	3.82	9.61	1.74	3.24	3.22 ± 3.08
Error Rate (%)	0.79	1.67	16.67	2.08	6.81 ± 8.54	5.36	5.36	5.00	6.79	9.64	6.43 ± 1.92	1.04	2.43	2.43	6.25	19.79	11.46	28.82	5.21	9.72	9.68 ± 9.24
Kappa	0.98	0.95	0.55	0.94	0.81 ± 0.23	0.89	0.89	0.90	0.86	0.81	0.87 ± 0.03	0.97	0.93	0.93	0.83	0.47	0.69	0.23	0.86	0.74	0.73 ± 0.24
Precision	0.98	0.98	0.84	0.97	0.93 ± 0.07	0.96	0.99	0.94	0.96	0.90	0.95 ± 0.03	0.98	0.97	0.97	0.93	0.81	0.88	0.71	0.95	0.90	0.90 ± 0.08
F1-Score	0.99	0.98	0.83	0.97	0.93 ± 0.08	0.94	0.94	0.95	0.93	0.90	0.93 ± 0.01	0.98	0.97	0.97	0.93	0.80	0.88	0.71	0.95	0.90	0.89 ± 0.09
MCC	0.98	0.97	0.78	0.97	0.91 ± 0.11	0.89	0.89	0.90	0.87	0.81	0.87 ± 0.03	0.98	0.96	0.96	0.91	0.74	0.85	0.61	0.93	0.87	0.86 ± 0.12
AUC	0.99	0.99	0.96	0.99	0.98 ± 0.01	0.98	0.98	0.99	0.98	0.97	0.98 ± 0.007	0.99	0.99	0.99	0.99	0.90	0.99	0.87	0.99	0.99	0.96 ± 0.04
Time (S)	2.53	1.40	1.35	1.41	1.39 ± 0.03	3.26	3.12	3.08	3.07	2.66	3.04 ± 0.22	3.38	2.78	3.25	2.78	2.79	2.80	2.78	2.78	2.78	2.89 ± 0.22

**Figure 7:**
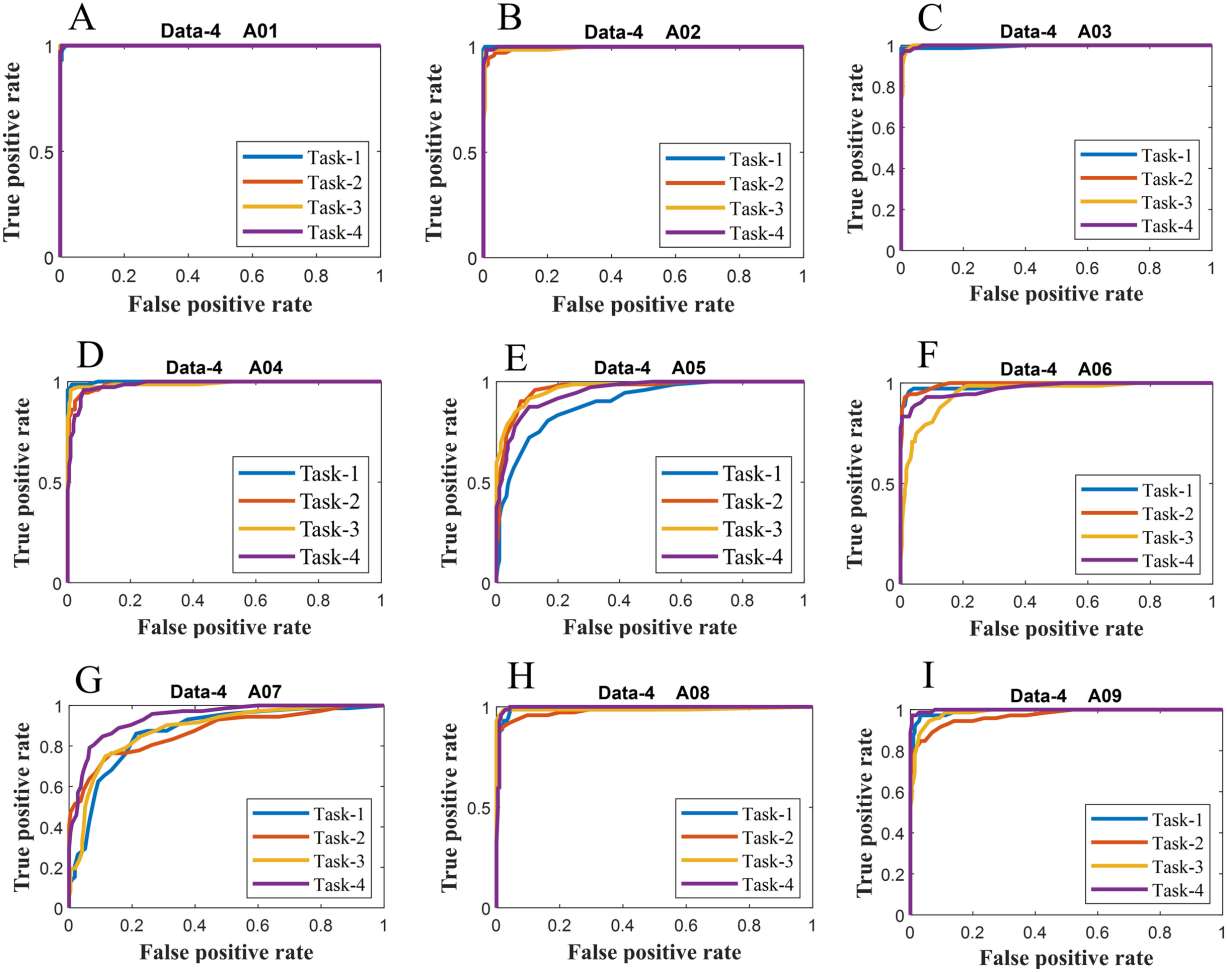
ROC of ERS-KNN classifier for (A) Data-4 A01 (B) Data-4 A02 (C) Data-4 A03 (D) Data-4 A04 (E) Data-4 A05 (F) Data-4 A06 (G) Data-4 A07 (H) Data-4 A08 and (I) Data-4 A09.

We have further evaluated the classifier performance in terms of ROC for all datasets utilised in the present study. Figure 6 illustrates ROC ERS- *k*-NN classifier for all subjects in data-1, data-2 and data-3, whereas Fig. 7 shows the ROC ERS-*k*-NN classifier for all subjects in data-4. In the case of data-1, task-1 and task-2 denote two different classes that are motor imagery movement of either left small finger or the tongue. In data-2, task-1, task-2, task-3, and task-4 denote four different classes which are left hand, right hand, tongue or foot movement imagery, respectively. The task-1 and task-2 in data-3 represent the left hand and the right-hand motor imagery, correspondingly. Finally, data-4 contains four different motor imagery tasks, namely the imagination of movement of the left hand (Task-1), right hand (Task-2), both feet (Task-3), and tongue (Task-4). In the case of four class problem (data-2 and data-4), the ROC has been plotted by following the binary classification approach, which is one vs all. The ROC plot in Figs. 6 and 7 reflect the similar performance of ERS-*k*-NN classifier as tabulated in Table 3. From the ROC curve, the reason behind the low accuracy of a classifier can be identified by analysing the true positive rate vs false-positive rate curve of every class. For example the classification accuracy of subject k6b in data-2 is very poor, and the reason for this poor accuracy can be interpreted by the ROC. The higher misclassification rate of task-2 reduces the accuracy of subject k6b in data-2.

**Figure 8:**
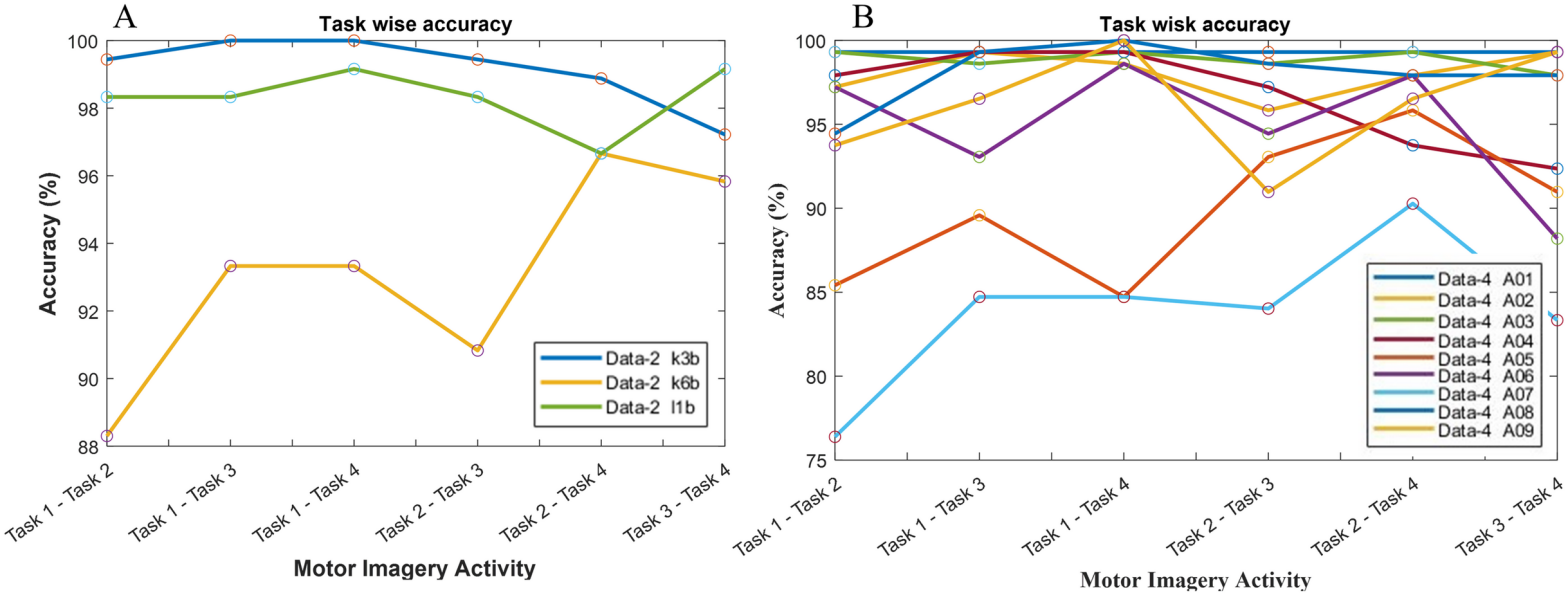
Classification accuracy with respect to binary class for (A) data-2 and (B) data-4.

The classification accuracy in terms of the binary class has also been computed for the investigation of the reason behind the poor classification accuracy in data-2 and data-4. Hence, a total of six binary classification models have been constructed since data-2 and data-4 consist of four class problems. In Fig. 8A, subject k3b shows excellent accuracy with all binary classification model except for ‘task 3-task 4’ model, which draws an accuracy of 97.22%. In the case of k6b, the performance of all the six models is comparatively poor. The lowest accuracy (88.3%) has been observed in ‘task 1-task 2’ model, whereas the highest accuracy (96.66%) has been observed in ‘task 2-task 4’ model. In the case of subject-l1b, the classification model for ‘task 2-task 4’ shows a comparatively poor accuracy (96.66%). However, the remaining five models exhibit very good accuracy. A similar investigation has been conducted in Fig. 8B for data-4.

**Figure 9:**
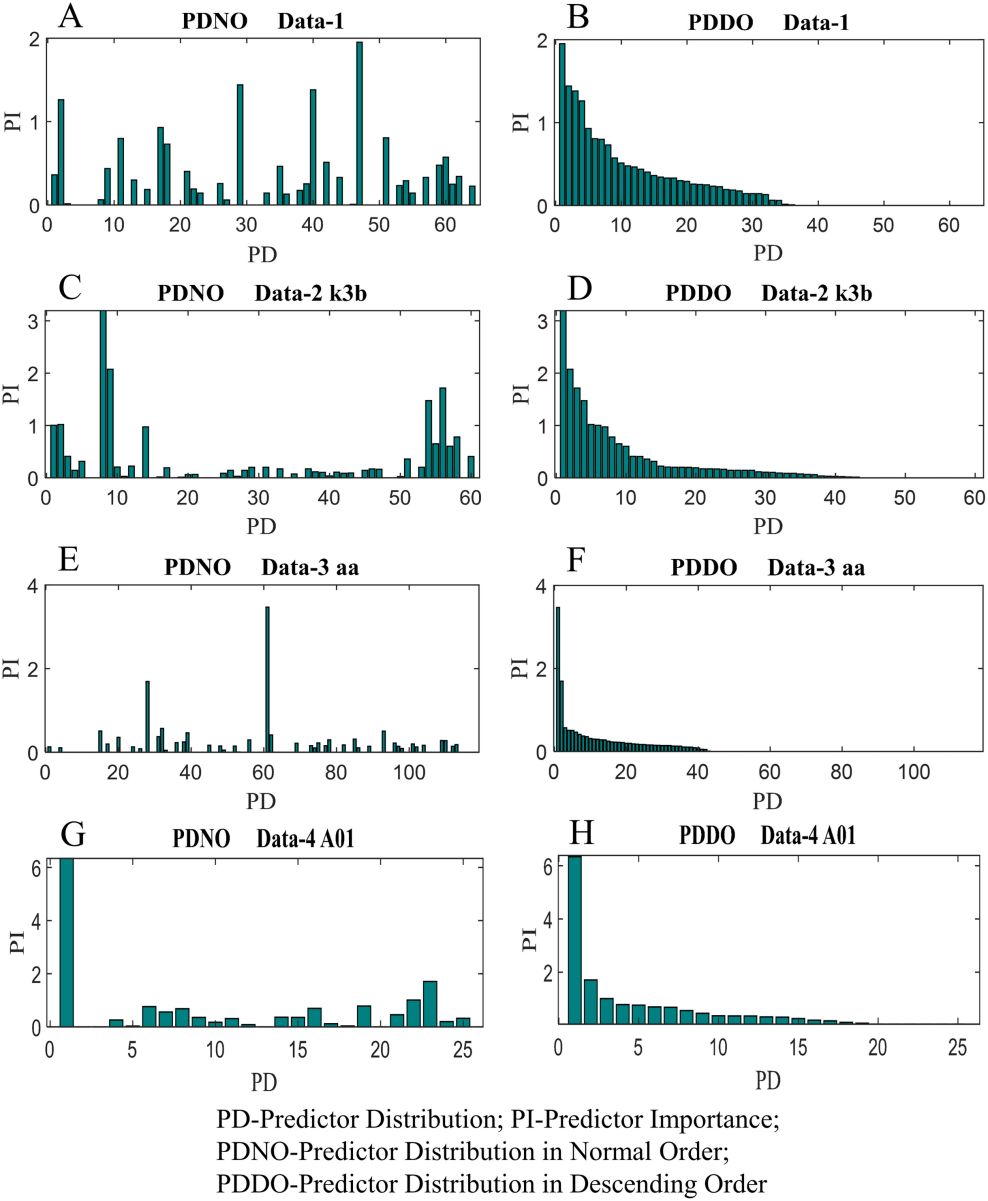
Distribution of predictor importance in the normal order and descending order for (A) PDNO Data-1 (B) PDDO Data-2 (C) PDNO Data-2 k3b (D) PDDO Data-2 k3b (E) PDNO Data-3 aa (F) PDDO Data-3 aa (G) PDNO Data-4 A01 and (H) PDDO Data-4 A01.

In this study, feature selection using random forest has also been investigated. The random forest algorithms select the most suitable feature sets based on OBB predictor importance by permutation. Figure 9 shows the distribution of predictor importance for all datasets utilised in this study. The first column in Fig. 9 presents the variation of predictor importance with predictor distribution in the normal order, whereas the second column in Fig. 9 illustrates the variation of predictor importance with predictor distribution in descending order. Basically, the predictors in Fig. 9 are the number of electrodes/channels. The number of electrodes in data-1, data-2, data-3 and data-4 are 64, 60, 118 and 25 respectively. Thus, the number of predictors in data-1, data-2, data-3 and data-4 are 64, 60, 118 and 25, respectively. From Fig. 9, it is evident that all the features are not equally important to contribute to the enhancement of the classification accuracy. The features which are less important, significantly increase the computational complexity and reduce the classification accuracy as well.

**Figure 10:**
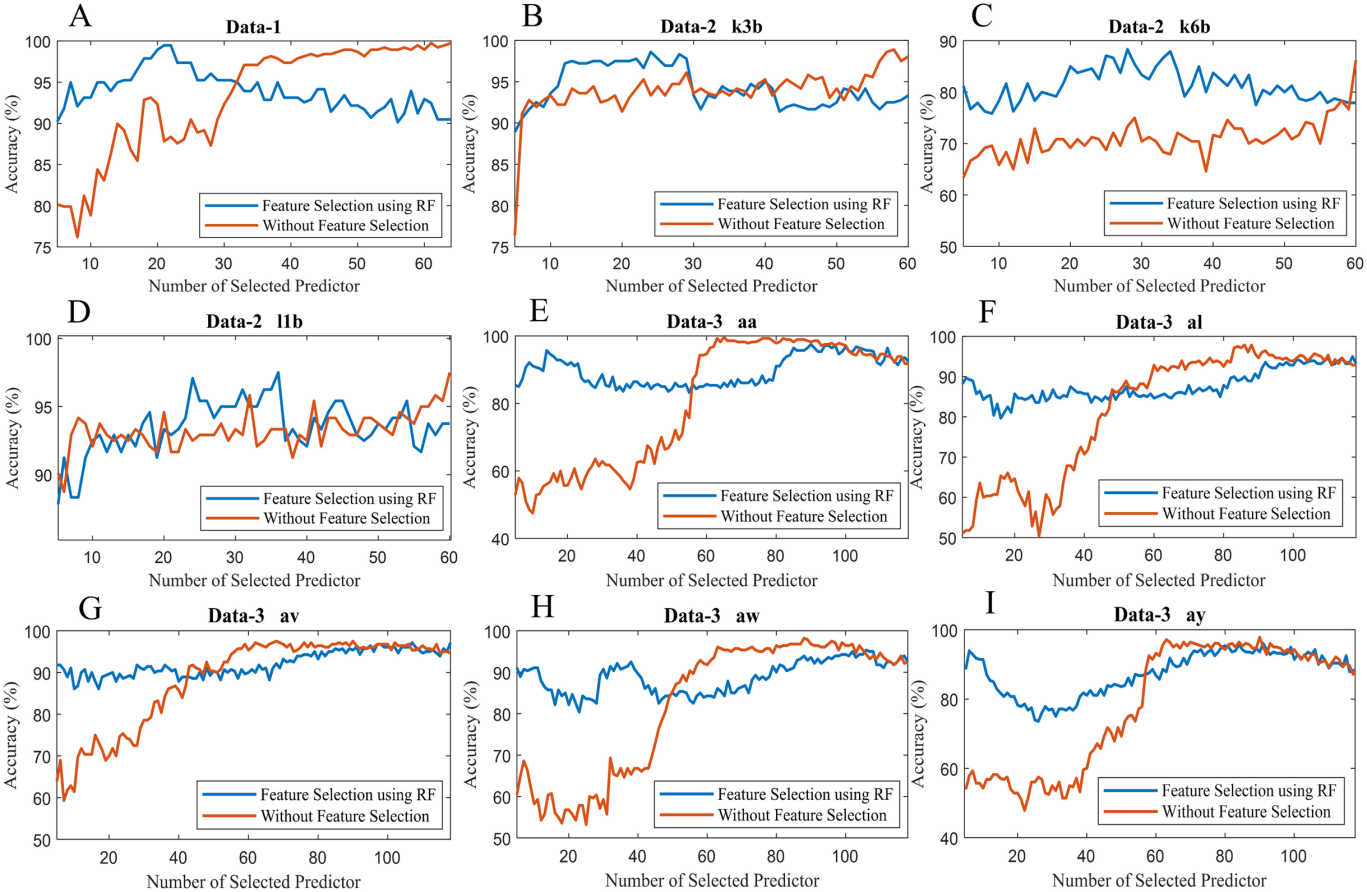
Classification accuracy with selected feature and without selected features of (A) Data-1 (B) Data-2 k3b (C) Data-2 k6b (D) Data-2 l1b (E) Data-3 aa (F) Data-3 al (G) Data-3 av (H) Data-3 aw and (I) Data-3 ay.

**Figure 11:**
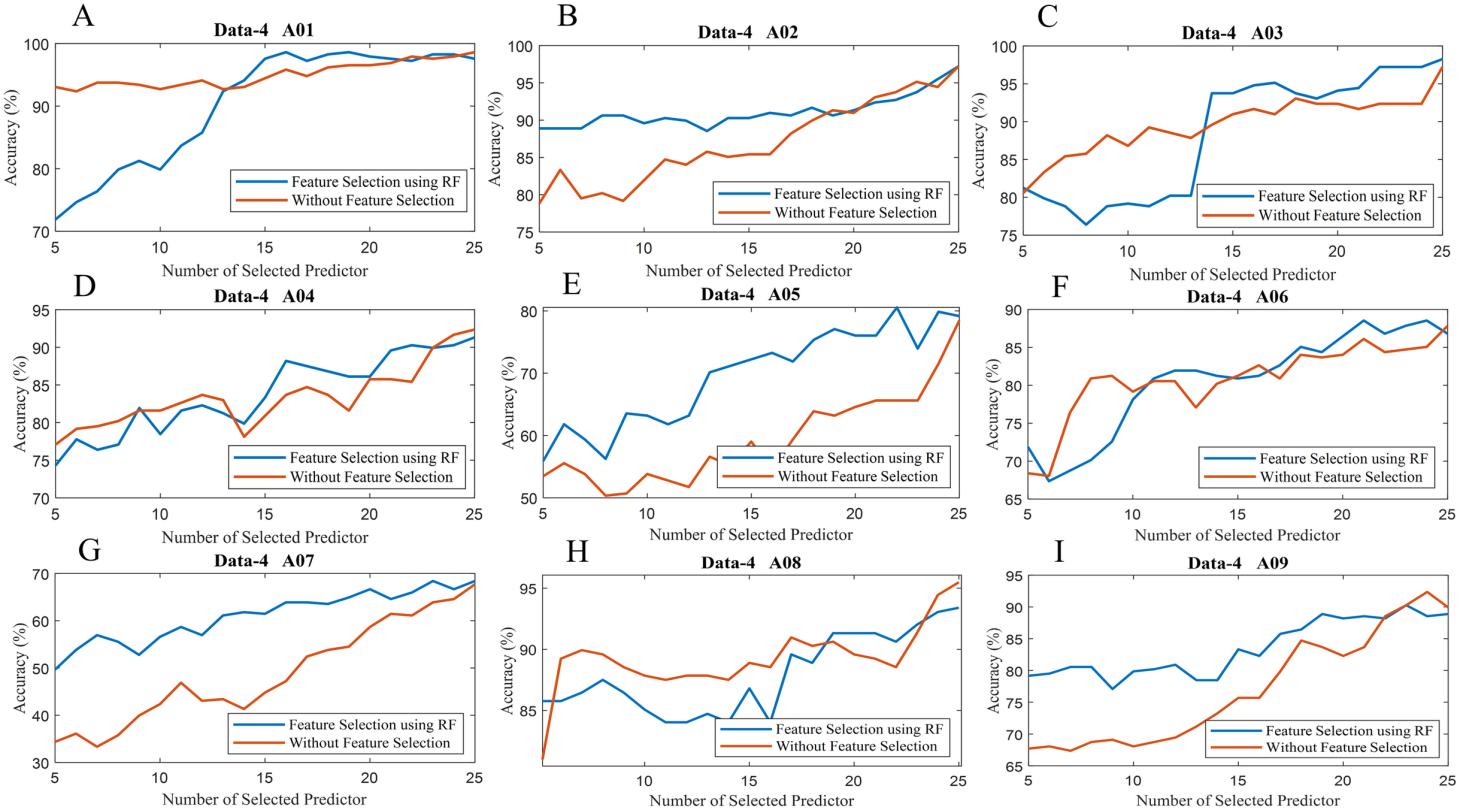
Classification accuracy with selected feature and without selected features of (A) Data-4 A01 (B) Data-4 A02 (C) Data-4 A03 (D) Data-4 A04 (E) Data-4 A05 (F) Data-4 A06 (G) Data-4 A07 (H) Data-4 A08 and (I) Data-4 A09.

To observe the effectiveness of the effect of identifying significant features, we have computed the classification accuracy of the models with the selected features and by considering all features (without selected features). Figure 10 illustrates the classification accuracy of both cases for data-1, data-2 and data-3. In the case of data-1, the highest accuracy, 99.21% has been achieved with 61 features out of 64 features when any feature selection algorithm has not employed. On the other hand, 98.94% accuracy has been obtained with only 21 features which are selected utilising random forest-based feature selection algorithm. In data-2 subject-k3b, 24 selected features using RF have achieved 98.61% accuracy, whereas 98.89% accuracy has been obtained without feature selection. Similarly, 28 and 36 selected features have been utilised to achieve the best accuracy (i.e. 88.33% and 97.50% respectively) in the case of subject-k6b and subject-l1b of data-2, respectively. In data-3, all the subjects have achieved almost maximum accuracy with a very small set of features which are selected by the RF. For example, subject-aa achieved 95.71% accuracy with only 16 selected features (using RF method) whereas, without feature selection process, around 62 features are utilised to obtain the same accuracy. Hence, it can be concluded that the RF-based feature selection algorithm can significantly improve the classification accuracy with fewer selected features. Similarly, Fig. 11 illustrates the classification accuracy with selected feature and without selected features for all subjects of data-4.

## Discussion

In this study, it is apparent that we have achieved better results by using the ensemble of random subspace *k*-NN, in comparison to other studies done on the same databases that have reported in the literature (see Tables 4–7). The results show that the proposed method provides reasonably accurate classification results even on challenging ECoG and EEG MI databases.

**Table 4:** Performance comparison of data-1 (BCI Competition III, Dataset 1).

Reference	Feature extraction	Feature selection	Classifier	Accuracy (%)
Erkan & Kurnaz (2017)	DWT	ADA	SVM	95
Islam et al. (2016)	CWT	PCA	SVM	92
Zheng et al. (2019)	MST	Cross-validation accuracy	SVM	95
Xu et al. (2016)	LBP		Gradient boosting (OLS)	95
Proposed Method	CSP	Random Forest	Ensemble of random subspace K-NN	99.21

**Table 5:** Performance comparison of data-2 (BCI Competition III Dataset IIIA).

Reference	Feature extraction	Feature selection	Classification	Accuracy (%)				
				k3b	k6b	l1b	Average	STD
Nguyen et al. (2018)	CSP	NA	FLS + PSO	91.8	75.6	92.2	86.5	9.47
Zhang et al. (2018)	CSP+TSGSP	NA	SVM	97.8	77.5	90.25	88.52	10.26
Selim et al. (2018)	CSP	AM-BA	SVM	98.89	77.50	83.33	86.57	11.06
She et al. (2018)	CSP	NA	FDDL-ELM	97.78	68.00	96.83	87.54	16.92
Jin et al. (2019)	R-CSP	CCS	SVM	98.9	80.0	96.7	91.9	10.33
Mishuhina & Jiang (2018)	FWR-CSP	NA	LDA	96.85	81.39	91.94	90	7.89
Proposed Method	CSP	Random Forest	Ensemble of random subspace K-NN	98.33	83.33	97.92	93.19	8.54

**Table 6:** Performance comparison of BCI data-3 (Competition III Dataset IVA).

Reference	Feature extraction	Feature selection	Classifiers	Accuracy (%)					Mean	STD
				aa	al	av	Aw	ay		
Chacon-Murguia, Olivas-Padilla & Ramirez-Quintana (2020)	CSP		SVM	73.21	83.93	64.8	75.00	77.38	74.86	6.93
Jin et al. (2020)	CSP	Bispectrum	LDA	82.1	93.9	73.6	93.6	93.2	87.28	9.12
Jin et al. (2019)	Regularized CSP	CCS	SVM	80.7	96.8	70.4	92.9	92.1	86.58	10.85
Guo et al. (2020)	FCCSP	mRMR	LDA	72.32	98.21	68.87	78.57	92.06	82.01	12.66
Su et al. (2020)	CSP		DCNMF	76.43	98.21	72.35	75.43	66.46	77.77	12.06
Park & Chung (2020)	FBCSP	CC	SVM	98.21	89.29	73.47	92.86	89.29	88.62	9.22
Selim et al. (2017)	RMS		LDA	69.64	89.29	59.18	88.84	86.90	78.77	13.65
Dai et al. (2018)			TKCSP	68.10	93.88	68.47	88.40	74.93	79.17	11.78
She et al. (2018)	FDDL		ELM	63.39	98.39	64.08	85.67	85.16	79.33	15.19
Singh, Lal & Guesgen (2019)			MDRM	81.25	100	76.53	87.05	91.26	87.21	9.07
Proposed Method	CSP	Random Forest		94.64	94.64	95.00	93.21	90.36	93.57	1.9

**Table 7:** Performance comparison of data-4 (BCI Competition IV Dataset IIA).

Reference	Feature Extraction	Feature Selection	Classifier	Classification Accuracy									Mean	STD	Kappa									Mean	STD
				A01	A02	A03	A04	A05	A06	A07	A08	A09			A01	A02	A03	A04	A05	A06	A07	A08	A09		
Uribe et al. (2019)	Correntropy	Wrapper	LDA or ELM												0.66	0.27	0.69	0.36	0.23	0.32	0.46	0.66	0.64	0.47	0.18
Rahman et al. (2020)	WPT	Rényi min-entropy	MLP-ANN	80	82	84	74	78	85	85	79	74	80.11	4.28											
Gaur et al. (2018)	Covariance matrix	–	RG	91.49	60.56	94.16	76.72	58.52	68.52	78.57	97.01	93.85	79.93	14.9	0.86	0.24	0.70	0.68	0.36	0.34	0.66	0.75	0.82	0.60	0.22
Rodrigues et al. (2019)	STR	FS	least squares	60	33	67	45	33	33	35	70	67	49	15	0.46	0.13	0.56	0.26	0.11	0.11	0.16	0.60	0.56	0.33	0.20
She et al. (2019)			HSS-ELM	92.10	70.26	94.40	80.86	71.41	78.31	92.10	89.22	92.67	84.59	9.54										0.79	
Zhang et al. (2019)	FBCSP		CNN-LSTM	89	69	92	82	84	68	95	90	92	84	9	0.87	0.59	0.90	0.76	0.82	0.66	0.95	0.89	0.89	0.81	0.12
She et al. (2020)			BGR-SSELM												0.79	0.46	0.81	0.59	0.38	0.43	0.81	0.82	0.81	0.65	0.18
Olivas-Padilla & Chacon-Murguia (2019)	DFBCSP		CNN	84.9	66.3	84.7	81.3	79.2	70.6	86.1	83.8	83.0	80.0	6.52	0.68	0.36	0.69	0.62	0.60	0.45	0.71	0.72	0.66	0.61	0.12
Liao et al. (2020)	Global spatial filters (FBCSP)		CNN	88.6	55.9	86.7	71.0	66.5	56.0	88.4	80.9	77.1	74.6	13.0	0.84	0.4	0.82	0.61	0.54	0.41	0.84	0.74	0.69	0.65	0.18
Proposed method				98.9	97.6	97.6	93.8	80.2	88.6	71.2	94.8	90.3	90.3	9.24	0.97	0.93	0.93	0.83	0.47	0.69	0.23	0.86	0.74	0.74	0.25

Most of the studies listed in Table 4 have achieved comparatively higher accuracy than the studies conducted by data-2, data-3 and data-4. There are some reasons behind this phenomenon, including quality of data, the variability of subjects and number of classes in the data. Table 4 has listed those studies which utilised two-class ECoG data (data-1) from a single subject. The signal to noise ratio of ECoG is higher than EEG, which may be another cause of higher accuracy of all studies in Table 4. Erkan & Kurnaz (2017) proposed an arc detection algorithm (ADA) to select the optimum channel. The features in terms of discrete wavelet transform (DWT) were extracted and then classified with the accuracy of 95%. In reference to Islam et al. (2016), the authors utilised CWT, PCA and SVM for feature extraction, selection and classification respectively on the same dataset and achieved a classification accuracy of 92%. Xu et al. (2016) proposed a hybrid feature extraction method consists of fractal measures and local binary pattern (LBP) operators. To classify the extracted feature, they employed gradient boosting of ordinary least squares (OLS) and achieved the classification accuracy of 95%. Zheng et al. (2019) proposed time-frequency based modified S-transform (MST) feature extraction algorithm, and then the extracted features were classified using SVM with 95% classification accuracy. The authors also selected the optimum channel using cross-validation accuracy and claimed that the proposed method significantly mitigate the algorithmic complexity. In comparison to other studies, our proposed method has achieved the classification accuracy of 99.21% with data-1.

To validate the proposed method more accurately, we have also employed the proposed method to the four classes EEG MI dataset (data-2). It is worth noting that several excellent studies tabulated in Table 5 have also been carried out to classify the similar MI dataset. Nguyen et al. (2018) proposed multi-class CSP based feature extraction method and fusion of fuzzy logic system (FLS) particle swarm optimisation (PSO) algorithm for the classification purpose. For the three subjects, they achieved an average accuracy of 86.5%. The studies in Jin et al. (2019), Zhang et al. (2018), Selim et al. (2018), She et al. (2018), Mishuhina & Jiang (2018) also utilised multi-class CSP to extract the feature although they employed a variety of feature selection and classification algorithm. The SVM has been utilised in different studies (Jin et al., 2019; Zhang et al., 2018; Selim et al., 2018) to classify this multi-class MI EEG data and achieved the average classification accuracy of 91.9%, 88.52% and 86.57%, respectively. Mishuhina & Jiang (2018) achieved a 90% accuracy to classify the same dataset by using LDA, whereas She et al. (2018) utilised FDDL-ELM and achieved the classification accuracy of 87.54%. Our proposed method have achieved a higher classification accuracy (93.19 ± 8.54%) as compared to the other methods. The accuracy could be higher than current achieved accuracy if the accuracy of subject-k6b was similar to the other subjects. We have noticed that the classification accuracy of subject-k6b dropped in almost all studies which demonstrate that subject-k6b contains artefact prone EEG.

The number of electrodes in data-1, data -2 and data-3, are 64, 60, and 118, respectively. It is worth noting that the classification accuracy may vary with the number of electrodes in the EEG. Table 6 tabulates other notable studies that have been carried out on data-3. Authors in Jin et al. (2020) classified data-3 by CSP-LDA approach. Moreover, they utilised bispectrum to select the channel and achieved the mean accuracy and standard deviation of 87.28% and 9.12%, respectively. Singh, Lal & Guesgen (2019) utilised minimum distance to Riemannian mean (MDRM) to classify data-3 and achieved an accuracy of (87.21 ± 9.07)%. The filter bank CSP features were classified by SVM in another study in Park & Chung (2020), and the recorded accuracy was (88.62 ± 9.22)%. In comparison with other studies in Table 6, the method proposed in the present study has achieved the highest accuracy viz. 93.57 ± 1.9%.

Subject dependency is another major issue in any BCI system. It is often anticipated that for any approach, a similar performance with respect to the same group of subjects is expected. The performance of the proposed method, that is ERS *k*-NN with feature selection via RF are assessed on data-1, data-2 and data-3, which consist of one, three and five subjects, respectively. To assess the subject independent performance, the proposed method has been evaluated on data-4, which consists of nine subjects. Table 7 has listed the summary of some recent studies where data-4 has been classified by means of different methods. Authors in Rahman et al. (2020) proposed wavelet packet transform (WPT)—MLP based approach to classify data-4. Besides this, the Rényi min-entropy has been implemented to identify the significant features, and an accuracy of 80.11 ± 4.28% was achieved. Zhang et al. (2019) proposed a hybrid deep learning architecture consists of CNN-LSTM to classify data-4 and achieved the accuracy of (84 ± 9)%. In another study, She et al. (2019) achieved an accuracy of (84.59 ± 9.54)% utilising hierarchical semi-supervised extreme learning machine (HSS-ELM). The accuracy attained through the present investigation is (90.3 ± 9.24)%, which is the highest in comparison to the studies listed in Table 7.

The random subspace approach is identical to bagging except that the attributes or features are randomly sampled, with substitution, for each learner. Informally, this allows individual learners to not over-focus on features that seem highly predictive in the training set but fail to be as predictive for points beyond that set. Thus, random subspaces are an appealing alternative of problems where the number of attributes is far greater than the number of training points, for instance, learning from EEG data. Moreover, the less computational complexity of this approach makes it a promising candidate for online BCI applications. According to the presented findings obtained from this study, we will highlight the followings:

- The CSP is an easy and far efficient feature extraction tool for MI-based BCIs, and the accuracy of the proposed architecture is better than state-of-the-art algorithms reported in the literature.
- In the case of machine learning algorithms, the ensemble random subspace K-NN can be confidently employed in the classification of EEG MI data due to its efficacy and robustness.
- Ensemble classifiers are trained separately from others and able to execute computational tasks parallelly.
- The training period of the proposed system is comparatively shorter than that of the comparable approaches, except for ensemble classifiers. The test time, however, is not only quick but also impartial of the training samples. Besides that, the presented approach is applicable to mobile devices.
- The limitation of ensemble learners is a large increase in the cost of computing. Nevertheless, the use of cloud-based parallel processing systems such as Hadoop can resolve the issue.

Although the proposed ERS- *k*-NN algorithm could significantly outperform other competing classification algorithms investigated, there are several limitations to this method. The major limitation is that the number of participants and the size of the dataset are restricted, resulting in a lack of sufficient persuasiveness. We can test the proposed method on more datasets in follow-up studies. Moreover, in this study, no artefact removal algorithm has been used, although the EEG contains highly artefact prone characteristics. Therefore, in the case of noisy EEG signals, the accuracy may be affected. Some preprocessing methods could be used to mitigate this problem, which warrants further investigation in the future. We shall also investigate the efficacy of the proposed method by considering frequency band and time window selections that could further enhance the performance of MI-based BCI. This multi-parameter optimisation problem will be investigated in our future study.

## Conclusions

The primary consumer of BCI technologies is the people who are severely affected by neuromuscular disorders, and commercialisation is the only way to spread this form of technology. Efficient signal processing architecture is one of the main issues that prevent or hampers the commercialisation of EEG based BCI system amongst the targeted users. In the signal processing phase, feature extraction and selection are non-trivial towards the attainment of reasonably-well classification accuracy. In this article, we have proposed an ensemble of random subspace *k*-NN based classification algorithm for binary as well as multi-class MI-based BCIs. Initially, the feature in terms of CSP has been extracted from MI EEG data. The significant features were selected based on the random forest algorithm. Finally, the proposed classification algorithm has been employed to calculate the classification accuracy. Experimental results suggest that the ensemble of random subspace *k*-NN algorithm can improve the performance of BCI systems and that the random forest-based feature selection method can further advance the performance of its classification accuracy. In a nutshell, the proposed approach is a promising candidate for improving the performance of MI-based BCI and a step closer to the implementation and realisation of such technology to the general masses.

## Supplemental Information

10.7717/peerj-cs.374/supp-1 Supplemental Information 1 Raw code.

## Additional Information and Declarations

### Data Availability

The following information was supplied regarding data availability: Raw MATLAB code is available in the Supplemental Files. Data is available at the BCI Competition website: - http://www.bbci.de/competition/iii/#datasets: Data-1: BCI Competition III, dataset I. - http://www.bbci.de/competition/iii/#datasets: Data-2: BCI Competition III, datasets IIIa. - http://www.bbci.de/competition/iii/#datasets: Data-3: BCI Competition III, datasets IVa. - http://www.bbci.de/competition/iv/#datasets: Data-4: BCI Competition IV, datasets 2a. In order to download the data, users need to do the following: Step 1: click the supplied web link. Step 2: After scrolling down, the user needs to submit their name and e-mail address. Step 3: After clinking the ‘I Agree’ button, an automatic e-mail containing access information to download the data will be sent to the user’s email address. Step 4: Using the access information, users can download the data. Publisher Note: The datasets used by the authors in this work were available at the stated URL when the manuscript was submitted and the reviewers had access to them in order to evaluate the work.
